# Translation and psychometric validation of the Medical Artificial Intelligence Readiness Scale (MAIRS-MS) for Chinese medical students

**DOI:** 10.1186/s12912-025-03852-w

**Published:** 2025-09-29

**Authors:** Xuancheng Chen, Yangyi Chen, Yuhuan Xie, Linan Cheng

**Affiliations:** 1 School of Nursing, Soochow University, Suzhou, 215006 People’s Republic of China; 2 School of Nursing, Lanzhou University, Lanzhou, 730000 People’s Republic of China; 3 School of Nursing, Wenzhou Medical University, Wenzhou, 325035 People’s Republic of China

**Keywords:** Medical students, Artificial intelligence, Readiness, Scale

## Abstract

**Background:**

With the rapid integration of artificial intelligence (AI) into medical education, assessing medical students’ readiness has become critical. This readiness encompasses not only familiarity with AI tools but also the ability to apply, evaluate, and ethically reflect on them. Despite international advances, China currently lacks a validated instrument to systematically evaluate medical students’ readiness for medical AI. Therefore, this study aimed to translate, culturally adapt, and evaluate the psychometric properties of the Medical Artificial Intelligence Readiness Scale (MAIRS-MS) for Chinese medical students.

**Methods:**

The MAIRS-MS was translated into Chinese following Brislin’s guidelines, with subsequent cultural adaptation informed by expert consultation. A pilot study was then conducted with 30 medical students to refine the Chinese version (C-MAIRS-MS). A cross-sectional survey was conducted among 516 undergraduate medical students from March to May 2025. The psychometric properties of the C-MAIRS-MS were evaluated through exploratory factor analysis (EFA), confirmatory factor analysis (CFA), Cronbach’s α coefficient, Spearman–Brown split-half reliability, and the intraclass correlation coefficient (ICC).

**Results:**

The C-MAIRS-MS included 22 items with scale content validity index (S-CVI) of 0.982. EFA extracted four factors explaining 65.274% of the total variance, with factor loadings ranging from 0.508 to 0.881. CFA results indicating that the revised 4-factor model was well fitted (χ²/df = 2.303, RMSEA = 0.071, CFI = 0.924, IFI = 0.925, and TLI = 0.912), with good structural validity. The Cronbach’s α coefficient, Spearman-Brown Split-half reliability, and ICC values for the C-MAIRS-MS were 0.935, 0.832, and 0.945, indicating the scale has good reliability.

**Conclusion:**

The C-MAIRS-MS demonstrated sound psychometric properties and provides a reliable tool to assess medical students’ readiness for medical AI. Beyond individual assessment, the scale can inform curriculum development, facilitate ongoing monitoring of students’ progress, and support the evaluation of AI-focused educational programmes, thereby offering educators valuable evidence to guide the design and refinement of AI-related training in medical education.

**Clinical trial number:**

Not applicable.

## Background

Artificial intelligence (AI) is an emerging technology with broad applications [1]. In the context of medical education, the integration of AI has shifted from a conceptual possibility to an urgent teaching necessity [2, 3]. While its transformative potential in clinical practice is widely acknowledged, the critical issue now lies in how medical students can be adequately prepared for AI integration [4]. This preparation requires not only familiarity with AI tools but also the ability to apply, evaluate, and ethically reflect on them within real-world medical contexts [5].

AI in medical education worldwide faces several pressing challenges. Key challenges include the absence of standardized curricula [6], limited practical implementation of AI tools [7], restricted access to AI training [8], inadequate student knowledge and competencies [9], a shortage of educators with AI expertise [10], and varying student attitudes and preparedness levels [11]. Moreover, AI-related courses and training often neglect ethical considerations and policy implications [1]. Addressing these gaps is critical to prepare students for AI integration in healthcare [12].

Medical AI readiness is defined as healthcare providers’ readiness to effectively employ AI applications. It reflects their ability to integrate AI with professional expertise to deliver clinical services across prevention, diagnosis, treatment, and rehabilitation [13]. However, existing studies have primarily examined medical students’ attitudes and perceptions of AI [14, 15], while their conceptual understanding, technical competencies, and practical skills related to AI remain insufficiently explored [9]. Furthermore, existing assessment tools such as scales and questionnaires focus mainly on attitudes and domain specific knowledge, neglecting the multidimensional nature of AI readiness [16]. Consequently, existing instruments tend to have a narrow scope, often measuring only isolated aspects of readiness and offering little guidance for curriculum reform or policy design. Such a limited perspective may fail to capture the full spectrum of skills required for medical students to use AI critically and responsibly.

Karaca et al. developed the Medical Artificial Intelligence Readiness Scale for Medical Students (MAIRS-MS), a psychometrically sound instrument with demonstrated reliability and validity [13]. This 22-item scale evaluates medical students’ medical AI readiness across four key dimensions: Cognition (8 items), Skills (8 items), Vision (3 items), and Ethics (3 items). Since its development, the MAIRS-MS has been successfully validated and applied across multiple international contexts, including Iran [17], Malaysia [18], Saudi Arabia [19], Turkey [20], and Jordan [21], consistently demonstrating both practical utility and measurement effectiveness.

Despite China’s rapid incorporation of AI in medical education [22], no validated instrument currently exists to systematically assess medical students’ readiness for AI. Without such a tool, it is difficult to identify students’ learning needs, monitor curricular effectiveness, and inform targeted educational reforms. This study aimed to translate and culturally adapt the MAIRS-MS into Chinese and to evaluate its reliability and validity. By providing a validated, multidimensional instrument covering cognitive, technical, visionary, and ethical competencies, the study seeks to support curriculum development, identify student learning needs, and inform future curriculum evaluation and educational planning in the context of AI integration in China.

## Methods

### Study design

This study is a cross-sectional and methodological design.

### Participant

This cross-sectional study was conducted from March to May 2025. As Zhejiang Province is a pioneering and highly developed region for AI in China, it provides a representative context for assessing AI readiness. A convenience sample of 516 undergraduate medical students was therefore recruited from two institutions in the province: a comprehensive research university in Hangzhou and a medical university in Wenzhou. These universities were selected for their diverse medical programs to ensure sample representativeness. An additional 30 qualifying medical students were separately recruited for test-retest reliability assessment (7-day interval), with this subgroup excluded from the primary cohort. Inclusion criteria required participants to be: (1) full-time undergraduate medical students; (2) psychologically stable with adequate communication capacity; and (3) willing to provide informed consent. Exclusion criteria comprised students on temporary leave (health-related, personal, or academic suspension).

### Instruments

The demographic section of the questionnaire collected general participant characteristics, including gender, age, academic year, major, voluntary major selection status, AI training exposure, AI knowledge level, perceived importance of AI curriculum, prior healthcare AI utilization, and healthcare AI usage frequency.

Chinese Version of the Medical Artificial Intelligence Readiness Scale for Medical Students (C-MAIRS-MS) [13]: The C-MAIRS-MS comprises 22-item instrument scored on a 5-point Likert scale ranging from “strongly disagree” (1) to “strongly agree” (5), with total scores ranging from 22 to 110. Higher scores indicate greater readiness for medical AI. The scale has demonstrated good reliability and construct validity (Cronbach’s α = 0.87). To ensure applicability among Chinese medical students, it underwent a rigorous process of translation, back-translation, cross-cultural adaptation, and psychometric evaluation.

### Translation procedure

After obtaining authorization from the original author via email, the scale was rigorously translated into Chinese following Brislin’s translation model [23]. First, two bilingual master’s students in nursing independently translated the original English version into Chinese, producing two preliminary versions (C1 and C2). The research team compared and analyzed C1 and C2, and after multiple rounds of discussion and revision, consolidated them into a single version (C3). Then, two nursing doctoral students with years of overseas study experience, who had not been exposed to the original scale, independently back-translated C3 into English, resulting in two versions (B1 and B2). The research team compared B1 and B2 against the original version, and after further discussion and revision, finalized the back-translated version (B3), which was submitted to the original author for review to ensure consistency in content, semantics, and format with the original scale. To ensure the scale’s professional rigor and cultural appropriateness, a panel of 15 experts was invited to evaluate the relevance of each item in the Chinese version. The panel consisted of 5 specialists in nursing education, 3 in medical education, 2 in nursing informatics, 2 in nursing psychology, 2 in artificial intelligence, and 1 in Chinese language and literature. Among the experts, 12 held doctoral degrees, 2 held master’s degrees, and 1 held a bachelor’s degree. Additionally, 7 experts held associate senior titles or higher. The mean age of the panel was 41.27 years (SD = 9.62), with a mean work experience of 15.47 years (SD = 11.68). The expert authority coefficient was 0.887. Item relevance was assessed using a 4-point Likert scale (1 = not relevant, 2 = somewhat relevant, 3 = relevant, 4 = highly relevant). The research team revised the scale based on expert feedback and developed a draft for pilot testing. Subsequently, a sample of 30 medical students (Excluded from the formal investigation) who met the inclusion criteria was selected to complete the revised draft of the C-MAIRS-MS. Based on their feedback and suggestions, further refinements were made to the item wording and structure. This process resulted in the final version of the C-MAIRS-MS.

### Data collection

The survey was administered via Wenjuanxing (https://www.wjx.cn/), a secure online survey platform. After obtaining institutional approval, the research team distributed the survey link through DingTalk, a widely used Chinese communication platform. Prior to participation, all respondents received detailed information about the study’s purpose, voluntary nature, and data anonymity, with written consent provided electronically. According to the sample size estimation method proposed by Kendall [24], the required sample size is typically ten times the number of items. With 22 items in this study and allowing for a 10% attrition rate, a minimum of 242 participants was needed. Additionally, to satisfy the requirements of confirmatory factor analysis, the sample size should to be at least 200 participants [25]. Therefore, the final target sample size was set at a minimum of 484 participants. Of the 545 questionnaires initially distributed, 516 were retained as valid after excluding 29 responses that either (a) were completed in less than three minutes or (b) displayed patterned responses indicative of inauthentic participation, thus meeting the sample size requirements.

### Statistical analysis

Item analysis and reliability and validity testing were conducted using SPSS version 25.0 (IBM Corp., Armonk, NY, USA) and AMOS version 24.0. Categorical data were presented as frequencies and percentages, while continuous data were expressed as means and standard deviations. Item analysis was performed using critical ratio (CR) method and Pearson correlation coefficients. Internal consistency reliability was assessed by Cronbach’s α coefficient and Spearman-Brown split-half reliability, and stability was evaluated via test-retest reliability. Validity assessment included content validity and construct validity. The significance level was set at α = 0.05.

### Project analysis

This study evaluated each item of the C-MAIRS-MS using both the correlation coefficient method and the CR method. For the correlation coefficient method, the correlation between each item score and the total scale score was examined. Items with a correlation coefficient ≥ 0.3 and statistical significance (*P* < 0.05) were considered to have good item-total correlation and overall discrimination and were retained; items with a correlation coefficient < 0.3 or lacking statistical significance (*P* > 0.05) were considered for deletion [26]. Additionally, the CR method involved by ranking participants’ total scale scores in descending order and selecting the top 27% and bottom 27% as the high and low-score groups, respectively [27]. Independent samples t-tests were conducted to compare item scores between these two groups. Items with a CR value < 3.00 or showing no statistically significant difference between groups (*P* > 0.05) were considered for deletion [26].

### Validity analysis

#### Content validity

This study involved 15 experts to evaluate the content validity of the scale. Using a 4-point Likert scale, the relevance of each item in the C-MAIRS-MS was assessed [28]. Item-level content validity index (I-CVI) and scale-level content validity index (S-CVI) were calculated. Generally, an I-CVI ≥ 0.78 and an S-CVI ≥ 0.80 indicate good content validity of the scale [28].

#### Construct validity

Exploratory Factor Analysis (EFA) and Confirmatory Factor Analysis (CFA) were conducted to evaluate the construct validity of the scale. A subset of the sample (*n* = 258) was randomly selected for EFA using principal component analysis with varimax rotation. Common factors with eigenvalues greater than 1 were extracted, and items with factor loadings below 0.40 or exhibiting cross-loadings were excluded. The suitability for EFA was confirmed by a Kaiser-Meyer-Olkin (KMO) value greater than 0.8 and a statistically significant Bartlett’s test of sphericity (χ², *P* < 0.05) [29]. Items with factor loadings above 0.5 and a cumulative variance contribution rate of at least 60% were considered to demonstrate good structural validity [30]. Subsequently, CFA was performed on the remaining 258 questionnaires using AMOS 24.0 to construct the CFA model. Multiple fit indices were used to further verify the model fit, including χ²/df, Root Mean Square Error of Approximation (RMSEA), Comparative Fit Index (CFI), Incremental Fit Index (IFI) and Tucker Lewis Index (TLI). A model was deemed to have good structural validity when χ²/df < 5, RMSEA < 0.08, CFI > 0.9, IFI and TLI > 0.9 [31].

### Reliability analysis

The scale’s reliability was evaluated through internal consistency and test-retest methods. Cronbach’s α coefficient and Spearman-Brown split-half reliability analyses demonstrated good internal consistency (α > 0.70) [32]. Test-retest reliability was assessed in a subsample of 30 nursing students with a 7-day interval between administrations. The intraclass correlation coefficient (ICC) results were categorized as: excellent (> 0.75), good (0.60–0.75), fair (0.40–0.59), or poor (< 0.40) [33].

### Ethics approval

This study was approved by the Ethics Committee of Soochow University in China (ethics number: SUDA2025H06). Prior to participation, each medical student signed an informed consent form. Participation was entirely voluntary, and participants were free to withdraw from the study at any time. To protect the privacy of participants, no personally identifiable information was collected, and all responses were anonymized.

## Results

### Cross-cultural adaptation results of the C-MAIRS-MS

The research team, in consultation with 15 experts, revised the wording of several questionnaire items to enhance the instrument’s applicability, while the total number of items remained unchanged. During the pilot survey, participants indicated that the items were clear and effectively captured the intended constructs, and therefore no substantive modifications were necessary. The expert-consulted revisions were as follows: Item 7, originally stated as “I can organize workflows in accordance with the logic of AI”, was revised to “I can organize workflows in accordance with the logic of AI (such as studying, researching, and translation, etc.)”; Item 8, originally stated as “I can express the importance of data collection, analysis, evaluation and safety; for the development of AI in healthcare”, was revised to “I can express the importance of data collection, analysis, evaluation, and safety for the development of AI in healthcare”; and Item 22, originally stated as “I can follow the legal regulations regarding the use of AI technologies in healthcare”, was revised to “I can follow regulations regarding the use of AI technologies in healthcare”. All revisions were reviewed and endorsed by the original authors, ensuring that the content and intent of the items were preserved.

### Demographic characteristics

The study included 516 medical students with predominantly female (71.1%, *n* = 367) and younger (≤ 20 years: 54.5%, *n* = 281). Academic distribution showed higher proportions of sophomores (31.2%, *n* = 161) and freshmen (28.7%, *n* = 148), with nursing students comprising 60.7% (*n* = 313) of the sample. A majority (78.1%, *n* = 403) reported voluntary program selection, with additional details presented in Table 1.

**Table 1:** Demographic data regarding medical students (*n* = 516)

Variables	*N*	%
**Sex**		
Male	149	28.88
Female	367	71.12
Age (years*)		
≤ 20	281	54.46
> 20	235	45.54
**Academic year**		
Freshman	148	28.68
Sophomore	161	31.21
Junior	82	15.89
Senior	74	14.34
Fifth	51	9.88
**Major**		
Nursing	313	60.66
Clinical medicine	67	12.98
Preventive medicine	136	26.36
**Voluntary major selection**		
Yes	403	78.10
No	113	21.90
**AI training received**		
Yes	83	16.09
No	433	83.91
**AI knowledge level**		
Proficient	17	3.29
Intermediate	155	30.04
Basic	319	61.83
None	25	4.84
**Perceived importance of AI curriculum**		
Important	424	82.17
General	77	14.92
Not important	15	2.91
**Prior healthcare AI utilization**		
Yes	343	66.47
No	173	33.53
**Healthcare AI usage frequency**		
0–1 times/week	182	35.27
2–4 times/week	202	39.15
≥ 5 times/week	132	25.58

### Item analysis

Correlation analysis revealed significant associations between each item and the total scale score (*r* = 0.638–0.824, *P* < 0.01), confirming all items’ validity in assessing medical students’ medical AI readiness. Consequently, all items were retained in the final scale [26]. CR analysis demonstrated values ranging from 13.697 to 23.437 for all items, with statistically significant differences between high and low-scoring groups (*P* < 0.05), indicating strong discriminative validity.

### Validity analysis

#### Content validity

Fifteen experts evaluated the content validity of the C-MAIRS-MS. The I-CVI ranged from 0.867 to 1.000, while the S-CVI was 0.982. Both indices exceeded the recommended thresholds (I-CVI ≥ 0.78, S-CVI ≥ 0.90), confirming excellent content validity of the scale.

### Exploratory factor analysis

The EFA was conducted on the 22 items of the C-MAIRS-MS. The KMO value was 0.920, and Bartlett’s test of sphericity was significant (χ² = 3524.859, df = 231, *P* < 0.001), indicating that the data were suitable for factor analysis. Principal component analysis with eigenvalues > 1 extracted four common factors, which together explained 65.274% of the total variance. All items had factor loadings greater than 0.5, and no item was deleted. Based on the content and alignment with the original scale, the four factors were named as follows: Factor 1 (Cognition) included six items (Q1 – Q6), Factor 2 (Ability) included ten items (Q7 – Q16), Factor 3 (Vision) included three items (Q17 – Q19), Factor 4 (Ethics) included three items (Q20 – Q22). Compared to the original MAIRS-MS, the structure of the C-MAIRS-MS remained consistent with four dimensions and 22 items. However, items Q7 and Q8, which were originally classified under the “Cognition” dimension in the original scale, were reassigned to the “Ability” dimension, possibly reflecting cultural specificity and contextual relevance within the Chinese setting. The detailed EFA results are presented in Table 2.

**Table 2:** Rotated component matrix of the scale (*n* = 258)

Item	F1	F2	F3	F4	Name of factors
Q16. I can choose the appropriate AI applications for the problems encountered in the healthcare	**0.737**	0.182	0.087	0.214	Ability
Q10. I can effectively and efficiently use AI technologies in healthcare services	**0.731**	0.172	0.111	0.247	
Q9. I can use AI-based information in combination with with my professional knowledge	**0.711**	0.255	0.288	0.172	
Q11. I can use the AI applications according to its purpose	**0.686**	0.152	0.379	0.100	
Q15. I can explain to the patient the AI applications used in healthcare services	**0.672**	0.182	0.139	0.185	
Q12. I can acquire, evaluate, use, share and create new knowledge through information and communication technologies	**0.632**	0.197	0.256	0.189	
Q14. I find it very valuable to apply AI to education, services and research purposes	**0.623**	0.085	0.440	0.050	
Q7. I can organize workflows in accordance with the logic of AI (such as studying, researching, and translation, etc.)	**0.608**	0.304	0.227	0.112	
Q13. I can explain how AI applications can provide a solution to a certain problem in the healthcare field	**0.589**	0.235	0.055	0.377	
Q8. I can express the importance of data collection, analysis, evaluation and safety; for the development of AI in healthcare	**0.508**	0.454	0.081	0.342	
Q4. I can define the basic concepts and terms of AI	0.241	**0.805**	-0.002	0.168	Cognition
Q1. I can define the basic contents of data science	0.121	**0.800**	0.131	0.058	
Q2. I can define the basic principles of statistics	0.110	**0.796**	0.094	-0.001	
Q3. I can explain how AI systems are trained	0.259	**0.709**	-0.044	0.210	
Q5. I can properly analyze the data obtained by AI in healthcare	0.388	**0.567**	0.089	0.306	
Q6. I can differentiate the functions and features of tools and applications related to AI	0.500	**0.520**	0.092	0.131	
Q21. I can act in accordance with medical ethical norms while using AI technologies	0.247	0.055	**0.881**	0.146	Ethics
Q20. I can use health data in accordance with laws, regulations and medical ethics norms	0.239	0.123	**0.877**	0.162	
Q22. I can follow regulations regarding the use of AI technologies in healthcare	0.222	-0.001	**0.863**	0.209	
Q17. I can explain the limitations of AI technology	0.259	0.129	0.170	**0.821**	Vision
Q19. I can foresee the opportunities and threats created by AI technology	0.239	0.202	0.168	**0.701**	
Q18. I can explain the strengths and weaknesses of AI technology	0.300	0.153	0.412	**0.618**	
Cumulative variance contribution rate	23.640%	40.452%	54.749%	65.274%	

### Confirmatory factor analysis

**Fig. 1:**
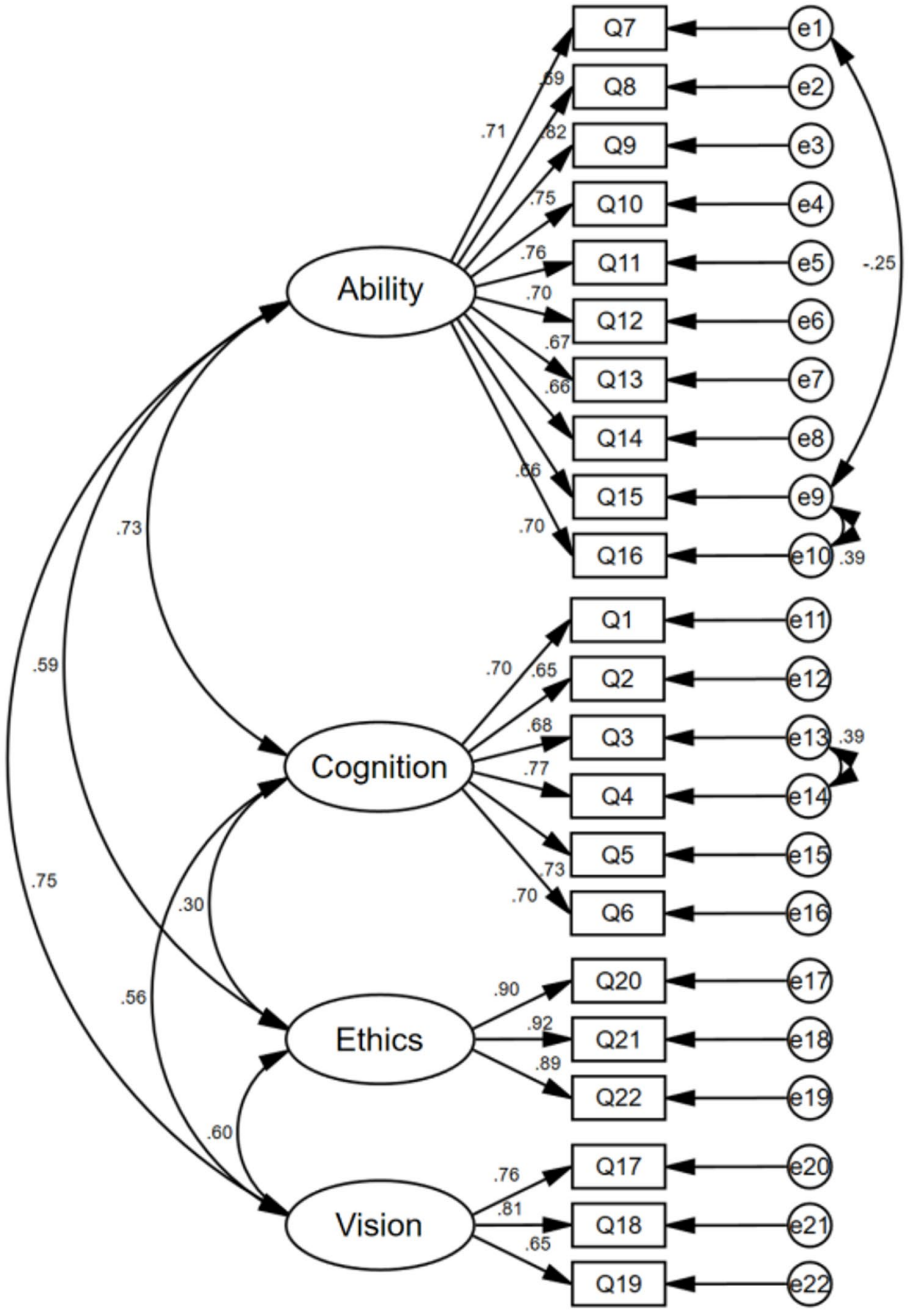
Standardized four-factor structural model of the C-MAIRS-MS (*n* = 258)

Guided by the modification indices (MI) [34], and with the dual aim of enhancing model fit while minimizing the risk of overfitting, the initial model was cautiously revised on three sequential occasions: first by correlating e1 and e9, followed by e9 and e10, and subsequently e13 with e14. In the final model (relative to the initial specification), the CFA results demonstrated an acceptable overall fit, with χ²/df = 2.303, RMSEA = 0.071, CFI = 0.924, IFI = 0.925, NFI = 0.874, GFI = 0.859, and TLI = 0.912. Detailed information of the CFA is presented in Fig. 1.

### Reliability analysis

The C-MAIRS-MS demonstrated excellent internal consistency and reliability in the EFA sample (*n* = 258), with a Cronbach’s α of 0.935 and a Spearman-Brown coefficient of 0.832, indicating strong internal consistency. All subscales demonstrated good reliability: Cognition (α = 0.863), Ability (α = 0.911), Vision (α = 0.778), and Ethics (α = 0.929). Test-retest reliability analysis revealed excellent stability, with an overall ICC of 0.928 (*P* < 0.05) and dimension-specific ICCs ranging from 0.892 (Ethics) to 0.921 (Ability).

## Discussion

Findings suggest that the C-MAIRS-MS is an appropriate instrument for assessing medical AI readiness among medical students. Psychometric evidence indicates strong validity and reliability, supporting its robustness in this context. The translation and cultural adaptation process adhered to Brislin’s model [35] and involved a multidisciplinary expert panel (*n* > 10), which ensured linguistic accuracy and conceptual equivalence with the original MAIRS-MS while optimizing item relevance for Chinese medical education settings. These adaptations enhanced the scale’s cultural appropriateness and clarity, supporting its practical use in curriculum development and evaluation within Chinese medical education.

In this study, EFA extracted four common factors consistent with the original structure. Specifically, in the Turkish [13] and Persian [17] of the MAIRS-MS, all items retained their original dimensional allocations, with Q7 and Q8 remaining under the “Cognition” dimension, reflecting an emphasis on theoretical understanding of AI. In contrast, in the C-MAIRS-MS, Q7 and Q8 were reclassified under the “Ability” dimension, highlighting a focus on practical application and skill demonstration in Chinese medical education [36, 37]. This difference may be influenced by cultural and teaching factors: in China, medical curricula often stress actionable competence and the direct application of knowledge, leading students to interpret AI-related tasks more in terms of capabilities than theoretical cognition. Despite this adjustment, the overall four-factor structure remains consistent between the two adaptations, suggesting that the core conceptual framework of the MAIRS-MS is stable across cultural contexts, while item-level alignment may reflect local educational priorities and cultural expectations. These modifications enhanced the scale’s cultural congruence and conceptual clarity. Moreover, compared with the original scale, the C-MAIRS-MS demonstrated higher factor loadings and a greater cumulative variance contribution rate in EFA, underscoring superior explanatory power. This advantage likely stems from refined item wording during cross-cultural adaptation and the relatively homogeneous, skills-oriented training of Chinese medical students, which minimized ambiguity and reinforced item–factor alignment. The C-MAIRS-MS’s alignment with the Chinese context appears to facilitate comprehension among students and may enable educators to more systematically assess readiness levels across multiple dimensions, potentially providing actionable insights for curriculum planning. Item-total correlations further indicate that each subscale meaningfully contributes to the overall assessment, allowing educators to pinpoint specific areas for instructional focus and targeted interventions [34].

Internal consistency and temporal stability were evaluated using Cronbach’s α coefficient and test–retest reliability, respectively. The Cronbach’s α of the C-MAIRS-MS were not only above the accepted threshold but also higher than those reported for the original version, demonstrating stronger internal consistency. The test–retest reliability similarly exceeded the standard threshold, indicating strong temporal stability. These strengths may be attributable to linguistic optimization, which reduced interpretive variability, and the curricular homogeneity of Chinese medical students, which enhanced response consistency across time. Overall, these findings suggest that the C-MAIRS-MS reliably captures students’ AI readiness over time, supporting longitudinal monitoring of learning outcomes and evaluation of educational interventions.

Content validity examines whether items adequately represent the target construct, while structural validity assesses the hypothesized dimensional framework [35]. High content and structural validity indicate that the scale effectively measures distinct dimensions of AI readiness, providing a reliable framework for curriculum developers to align course objectives with measurable competencies.

CFA was conducted to verify the hypothesized factor structure of the C-MAIRS-MS, with results demonstrating excellent model fit that were overall stronger than those reported in the original validation study, suggesting that the C-MAIRS-MS exhibits superior structural validity in the Chinese context. This improvement likely reflects the reallocation of certain items (e.g., Q7 and Q8) into dimensions more consistent with Chinese teaching practices, thereby enhancing structural coherence and cultural applicability. Psychometric evidence indicates strong discriminant validity, as evidenced by significant score differences between high and low-performing groups. Furthermore, all subscales showed strong convergent validity with the total score. These findings indicate that the four-dimensional framework captures distinct yet interrelated components of medical AI readiness, which could make it a practical tool for guiding curriculum development, monitoring student progress, and evaluating AI-focused educational programmes.

In summary, the C-MAIRS-MS provides a brief (< 5 min), multidimensional instrument (Cognition, Ability, Vision, and Ethics) that can be directly applied to curriculum development, student monitoring, and programme evaluation. Specifically, the cognition subscale can inform the integration of AI-related knowledge into curricula, the ability subscale can guide skills training and competency development, the vision subscale can support the cultivation of critical and anticipatory thinking in emerging AI contexts, and the ethics subscale can highlight the importance of legal and moral education. In practice, the scale can help identify potential curricular gaps, monitor students’ developmental trajectories over time, and evaluate the effectiveness of AI-focused educational programmes, thereby supporting evidence-informed educational planning and continuous improvement. Educators are advised to interpret scores alongside qualitative and contextual information to avoid overemphasis on measurable competencies or misinterpretation as definitive indicators of ability. Psychometric evidence indicates strong discriminant and construct validity, suggesting that the scale is an efficient and reliable measure within Chinese medical education contexts. Its performance is at least comparable to, and in some respects superior to, that of the original version, benefiting from careful cross-cultural adaptation and alignment with local teaching characteristics. While the scale demonstrates strong psychometric properties within classical validity frameworks, its potential consequences of application and broader social implications merit careful consideration from a modern validity perspective, as articulated by Messick and Kane [38, 39].

### Limitations

This study has several limitations. The use of a convenience sample from two universities in Zhejiang Province, while representative of a technologically advanced region, may limit the generalizability of the findings to other provinces or educational settings. In addition, the study specifically included undergraduate medical students from disciplines such as nursing, preventive medicine, and clinical medicine, but did not incorporate postgraduate students or practicing clinicians. Thus, the validity of the C-MAIRS-MS for these groups remains unestablished. Moreover, the cross-sectional design based on self-reported measures introduces the possibility of common method bias. Although the scale shows strong psychometric properties within the classical validity framework, this study did not examine additional forms of validation such as longitudinal stability, predictive validity, or social impact analysis. Future research should aim to include more diverse and representative samples, incorporate longitudinal designs, and examine predictive utility and broader consequential validity within real-world educational and clinical environments.

## Conclusions

This study validated the psychometric properties of the C-MAIRS-MS, offering medical educators a novel, domain-specific tool to assess students’ readiness for medical AI. Comprising 22 items across four dimensions, the C-MAIRS-MS demonstrated strong reliability and validity, indicating its potential use as a practical instrument for informing curriculum development, monitoring students’ progress, and evaluating the effectiveness of AI-focused educational programmes. By providing a structured, evidence-based approach to assess AI competencies, the C-MAIRS-MS uniquely enables educators to identify curricular gaps, support targeted skill development, and integrate AI readiness systematically into medical education. However, using this scale may have both intended and unintended consequences. Intended benefits include identifying curricular gaps, guiding skill development, and supporting evidence-informed educational planning. Potential unintended consequences could involve overemphasis on measurable AI competencies or misinterpretation of scores as definitive indicators of competence. Educators should interpret results alongside other qualitative and contextual information to ensure balanced educational decisions.

## Data Availability

The datasets used and/or analyzed during the current study are available from the corresponding author on reasonable request.

## Abbreviations

MAIRS-MS: Medical Artificial Intelligence Readiness Scale for Medical Students
SPSS: Statistical Product and Service Solutions
AMOS: Analysis of moment structure
KMO: Kaiser-Meyer-Olkin
EFA: Exploratory factor analysis
CFA: Confirmatory factor analysis
I-CVI: Item-level content validity index
S-CVI: Scale-level content validity index
ICC: Intraclass correlation coefficient
RMSEA: Root mean square error of approximation
CFI: Comparative fit index
TLI: Tucker Lewis index
NFI: Normed fit index
GFI: Goodness-of-fit index
IFI: Incremental fit Index
